# Vitamin D Receptor Polymorphisms and the Effect of Vitamin D Supplementation on Diabetes Risk Among Adults With Prediabetes

**DOI:** 10.1001/jamanetworkopen.2026.7332

**Published:** 2026-04-23

**Authors:** Bess Dawson-Hughes, Gordon S. Huggins, Jason Nelson, Ellen Vickery, Sarah N. Powers, Anastassios G. Pittas

**Affiliations:** 1 Jean Mayer USDA Human Nutrition Research Center on Aging at Tufts University, Boston, Massachusetts; 2 Division of Cardiology, Tufts Medical Center, Boston, Massachusetts; 3 Division of Endocrinology, Tufts Medical Center, Boston, Massachusetts

## Abstract

**Importance:**

Achieving and maintaining a serum 25-hydroxyvitamin D (25[OH]D) level of 40 ng/mL or higher, compared with 20 to 30 ng/mL, may lower diabetes risk among adults with prediabetes. It is not known whether a genetically defined subgroup is more likely to experience benefits from targeting higher 25(OH)D levels with vitamin D**_3_** supplementation.

**Objective:**

To assess the role of common polymorphisms of the vitamin D receptor (VDR) in the association between supplementation with 4000 IU/d of vitamin D**_3_** and the risk of diabetes among adults with prediabetes.

**Design, Setting, and Participants:**

This genetic association study conducted a VDR genotype analysis of 3 common polymorphisms—ApaI, BsmI, and FokI—among 2098 participants in the Vitamin D and Type 2 Diabetes (D2d) clinical trial with available intratrial 25(OH)D levels and genotyping. The D2d trial was conducted from October 1, 2013, to November 28, 2018, with statistical analysis performed from January 3 to November 30, 2025.

**Exposures:**

Receipt of 4000 IU/d of vitamin D**_3_** vs placebo for a median of 2.5 years (IQR, 1.8-3.5 years).

**Main Outcomes and Measures:**

In the discovery phase analysis among 1903 participants with available data, the risk of diabetes across different intratrial mean 25(OH)D levels in association with the VDR polymorphisms was examined. This was followed by a test phase analysis examining the response to vitamin D**_3_** supplementation on incident diabetes among 2098 participants according to the ApaI genotypes.

**Results:**

Of 2098 adults with prediabetes (mean [SD] age, 60.2 [9.9] years; 1169 men [55.7%]) in the test phase analysis, 618 with ApaI AA alleles exhibited no response to treatment with vitamin D**_3_** (hazard ratio [HR], 1.02 [95% CI, 0.72-1.44]; models adjusted for study site, race and ethnicity, sex, baseline age, body mass index, usual physical activity, statin use, and intratrial weight change). In contrast, 1480 participants with ApaI AC and CC genotypes showed a 19% decrease in the risk of diabetes with vitamin D**_3_** (HR, 0.81 [95% CI, 0.66-0.99]).

**Conclusions and Relevance:**

This genetic association study of adults with prediabetes suggests that diabetes risk reduction after supplementation with 4000 IU/d of vitamin D**_3_** was restricted to participants carrying the AC and CC alleles of the ApaI polymorphism. These findings support the potential role of ApaI genotyping in identifying individuals most likely to experience benefits from high-dose vitamin D**_3_** treatment to reduce diabetes risk.

## Key Points

**Question:**

How do polymorphisms in the vitamin D receptor influence the effect of vitamin D**_3_** supplementation on diabetes risk among adults with prediabetes?

**Findings:**

In this genetic association study using data from the D2d study, 618 adults with the AA genotype of the ApaI polymorphism (rs797523) did not respond to treatment with 4000 IU/d of vitamin D_3_, compared with placebo (hazard ratio [HR], 1.02 [95% CI, 0.72-1.44]). In contrast, 1480 adults with AC and CC genotypes had a reduced risk of diabetes (HR, 0.81 [95% CI, 0.66-0.99]).

**Meaning:**

This study suggests that ApaI genotyping may identify adults with prediabetes who experience benefits from high-dose vitamin D**_3_** supplementation to lower their risk of developing diabetes.

## Teaser

This genetic association study assesses the role of common polymorphisms of the vitamin D receptor in the association between supplementation with 4000 IU/d of vitamin D**_3_** and the risk of developing diabetes among adults with prediabetes.

## Introduction

Improving vitamin D status may reduce diabetes risk, particularly among individuals with prediabetes who are at high risk of progression to type 2 diabetes. To test this hypothesis, 3 vitamin D and diabetes prevention randomized clinical trials have been conducted, enrolling participants with prediabetes and using progression to diabetes or regression to normoglycemia as key outcomes. In an individual-participant meta-analysis of these trials, vitamin D reduced the risk of developing diabetes among adults with prediabetes.^1^ In 1 of these trials, the Vitamin D and Type 2 Diabetes (D2d) study in the US, participants with prediabetes were randomized to receive 4000 IU of vitamin D_3_ or placebo daily and were followed up for a median of 2.5 years (IQR, 1.8-3.5 years) for incident diabetes.^2^ Although the primary outcome did not reach statistical significance in the intent-to-treat analysis (hazard ratio [HR], 0.88 [95% CI, 0.75-1.04]), further analyses revealed that the effect of vitamin D**_3_** depended on the achieved intratrial serum 25-hydroxyvitamin D (25[OH]D) levels. Specifically, participants who maintained intratrial 25(OH)D levels of 40 to 49 ng/mL (to convert to nanomoles per liter, multiply by 2.496) during follow-up had a 45% risk reduction, and those who maintained intratrial 25(OH)D levels of 50 ng/mL or more had a 66% risk reduction in incident diabetes compared with those who maintained intratrial 25(OH)D levels of 20 to 29 ng/mL.^3,4^ This finding was replicated in the individual-participant meta-analysis.^1^

The effect of vitamin D supplementation on diabetes risk may be modified by genetic variability in the vitamin D receptor (VDR). In the UK Biobank, among adults of European ancestry with prediabetes, the baseline 25(OH)D level was inversely associated with risk of developing type 2 diabetes over 14 years of follow-up, and those carrying the rs1544410 (BsmI) TT genotype had greater risk reduction at higher 25(OH)D levels compared with those with TC or CC genotypes.^5^ A similar trend was observed for rs731236 (TaqI) but not for rs7975232 (ApaI) or rs2228570 (FokI). The biological plausibility for these findings is supported by evidence that the VDR is expressed in pancreatic β cells, influencing insulin secretion and glucose homeostasis.^6,7^

We hypothesized that VDR gene variants modify the association between achieved intratrial 25(OH)D level and diabetes risk and may modify the effect of vitamin D**_3_** supplementation on the risk of developing diabetes. The aims of this genetic association study were to examine 3 common VDR polymorphisms in the D2d trial and evaluate: (1) whether these polymorphisms were associated with reduced diabetes risk among participants who achieved higher intratrial mean 25(OH)D levels (discovery phase) and (2) whether participants’ VDR genetic profile modified the response to vitamin D**_3_** supplementation compared with placebo (test phase).

## Methods

### Overview of the D2d Study

The D2d study was a multisite randomized, double-blind, placebo-controlled clinical trial conducted from October 1, 2013, to November 28, 2018, to test whether 4000 IU/d of vitamin D**_3_**, compared with placebo, lowered incident diabetes among adults with prediabetes. The median follow-up period was 2.5 years (IQR, 1.8-3.5 years). The study design has been published^8^ and is briefly summarized here. The study was approved by the institutional review board at each clinical site (eTable 2 in Supplement 1), and all participants provided written informed consent. This first reported genetic association study adheres to the Strengthening the Reporting of Genetic Association Studies (STREGA) reporting guideline.^9^

Eligible participants met at least 2 of 3 glycemic criteria for prediabetes as defined by the 2010 American Diabetes Association guidelines^10^: fasting plasma glucose (FPG) level of 100 to 125 mg/dL (to convert to millimoles per liter, multiply by 0.0555); plasma glucose level of 140 to 200 mg/dL 2 hours after a 75-g oral glucose load (2hPG); hemoglobin A_1c_ (HbA_1c_) level of 39 to 47 mmol/mol (5.7%-6.4%) (to convert to proportion of total hemoglobin, multiply by 0.01); and not meeting any of the criteria for diabetes. Other inclusion criteria were age 30 years or older. Exclusion criteria included use of diabetes or weight-loss medications, hyperparathyroidism, nephrolithiasis, hypercalcemia, and bariatric surgery. Participants were asked to refrain from using diabetes-specific and/or weight-loss medications during the study and to limit the use of vitamin D outside of the study to 1000 IU/d from all supplements.

Glycemic status was assessed annually with FPG, HbA_1c_, and 2hPG levels and semiannually with FPG and HbA_1c_ levels. If at least 2 of the glycemic measures met the American Diabetes Association thresholds for diabetes (FPG level ≥126 mg/dL, 2hPG level ≥200 mg/dL, or HbA_1c_ level ≥6.5%),^10^ the participant was considered to have met the diabetes outcome.

### Conceptual Approach to the Current Analyses

#### Discovery Phase

In this phase, we assessed whether VDR polymorphisms played a role in the association between the intratrial mean 25(OH)D level and incident diabetes. Eligibility for this analysis was the same as that of a prior analysis in the D2d study that examined the effect of the intratrial mean 25(OH)D level on incident diabetes among participants with available data.^3^ The discovery analysis was restricted to 1903 participants with available intratrial 25(OH)D levels and genotype (for ≥1 polymorphisms). For each participant, intratrial vitamin D**_3_** exposure was calculated as a cumulative mean of all available yearly serum 25(OH)D values before the occurrence of new-onset diabetes, the start of a diabetes or weight-loss drug, or last follow-up, as previously described.^3^ Consistent with prior work in the D2d study, we chose as the intratrial vitamin D**_3_** referent 20 to 29.9 ng/mL, the lower part of the adequate range of 20 to 50 ng/mL based on the National Academy of Medicine, as described previously.^3^ The outcome variable was incident diabetes.

#### Test Phase

In the test phase, we replicated the primary analysis of the D2d study and examined whether the treatment with 4000 IU of vitamin D_3_ daily vs placebo for incident diabetes differed across subsets of participants classified as *potential responders* or *potential nonresponders*. Based on the discovery analyses, participants were classified as potential nonresponders to vitamin D**_3_** supplementation if they had alleles that were not associated with decreased risk of diabetes at higher achieved 25(OH)D levels. Conversely, participants were classified as potential responders if they had alleles associated with reduced risk of diabetes at higher intratrial 25(OH)D levels. The test analyses included all participants with available genotype data (some of whom did not have cumulative 25[OH]D levels).

### Biochemical and Genetic Measurements

Serum 25(OH)D levels were measured in stored fasting serum samples collected at baseline and at months 12, 24, 36, and 48 by liquid chromatography–tandem mass spectrometry with calibrators traceable to the National Institute of Standards and Technology and validated through a quarterly proficiency testing program administered by the Vitamin D External Quality Assessment Scheme (DEQAS, London, UK).^11,12^ The coefficient of variation of this assay is 5% to 8%.

Blood samples collected in PaxGene tubes were stored in a −80 °C freezer before 200 µL from each sample was used to purify genomic DNA using the QIAmp 96 DNA QIACube HT kit (catalog number 51331; Qiagen). TaqMan genotype reactions included 10 µL of 40× diluted DNA, 10 µL of Genotyping Master Mix (catalog number 4371355; Applied Biosystems), and 0.25 µL of assay reagent (catalog numbers: C_8716062 for BsmI [rs1544410], C_28977635 for ApaI [rs7975232], and C_12060045 for Fok1 [rs2228570]). Each TaqMan genotype assay was performed on a QuantStudio3 (Applied Biosystems), and genotypes were assigned using the QuantStudio Design and Analysis Software, version 1.4. Genotype call rates were in excess of 98.9%.

### Statistical Analysis

Statistical analysis was performed from January 3 to November 30, 2025. The distribution of VDR alleles across the achieved 25(OH)D categories was assessed by Pearson χ^2^ tests. The discovery analyses identified risk of diabetes at different intratrial mean 25(OH)D levels by alleles of the 3 polymorphisms. Time to event was defined as the time from randomization to the occurrence of incident diabetes. The follow-up time was the time from randomization until the occurrence of incident diabetes, death, withdrawal, or the last follow-up visit. Because this per-protocol analysis aims to capture the associations of the intervention using data obtained while participants were receiving active treatment and prior to the introduction of rescue therapies (and to align with prior analyses in the D2d study^3^), follow-up was additionally censored at the time a participant stopped trial pills, started a diabetes or weight-loss medication, or took outside-of-study supplemental vitamin D above the study limit of 1000 IU per day. Cox proportional hazards regression models for right-censored failure time outcomes were used to estimate HRs and 95% CIs for new-onset diabetes. Based on clinical rationale and to stay consistent with prior D2d analyses,^2^ regression analyses were adjusted for study site; self-reported race (Asian, Black, White, or other [American Indian or Alaska Native, Native Hawaiian or Other Pacific Islander, and other race]), ethnicity (Hispanic or non-Hispanic), and sex; baseline values of age, body mass index (calculated as weight in kilograms divided by height in meters squared), usual physical activity, and statin use; and intratrial weight change. Race was included as a proxy for skin pigmentation, which influences vitamin D physiology,^13^ and because it is associated with diabetes risk. Ethnicity was included because of its association with diabetes risk and because reporting of ethnicity is required in National Institutes of Health–funded research.

The test analyses involved adjusted Cox proportional hazards regression models to calculate the HRs for new-onset diabetes between the treatment groups among the nonresponders and responders. The models included treatment group assignment as the main variable for the nonresponder and responder cohorts. Covariates were the same as those used in the discovery analyses. These models showed no evidence of violation of the proportional hazards assumption. The Kaplan-Meier estimator was used to display the incidence of type 2 diabetes by treatment assignment over time in nonresponders and responders. Analyses were conducted using SAS software, version 9.4 M7 (SAS Institute Inc). A prespecified α level of .05 was used for all statistical tests to determine significance.

## Results

### Discovery Analysis Phase

The baseline characteristics of the 1903 participants (mean [SD] age, 60.6 [9.7] years; 1056 men [55.5%] and 847 women [44.5%]; 100 Asian [5.3%], 435 Black [22.9%], 1319 White [69.3%], and 49 other race or ethnicity or not provided [2.6%]; 172 Hispanic or Latino [9.0%]) in the discovery analysis who had available mean intratrial 25(OH)D values and genotype data are shown in Table 1, by 25(OH)D category and overall. These characteristics were generally balanced. The intratrial mean (SD) 25(OH)D levels ranged across the categories from 15.4 (3.3) to 59.9 (8.2) ng/mL. The mean (SD) weight change during the trial was minimal (−0.1 [5.5] kg) and did not differ significantly by 25(OH)D category. The distribution of alleles of the ApaI, BsmI, and FokI VDR polymorphisms did not differ significantly across the 25(OH)D categories.

**Table 1.** Baseline Characteristics, Weight Change, and VDR Alleles by Intratrial Mean Serum 25(OH)D Category Among Participants in the Discovery Analysis

Characteristic	Intratrial mean serum 25(OH)D category, ng/mL					Total (N = 1903)
	<20 (n = 208)	20-29.9 (n = 403)	30-39.9 (n = 525)	40-49.9 (n = 365)	≥50 (n = 402)	
Age, mean (SD), y	55.5 (10.4)	59.3 (10.1)	61.6 (9.6)	61.1 (9.4)	62.7 (8.3)	60.6 (9.7)
Sex, No. (%)						
Female	81 (38.9)	181 (44.9)	226 (43.0)	147 (40.3)	212 (52.7)	847 (44.5)
Male	127 (61.1)	222 (55.1)	299 (57.0)	218 (59.7)	190 (47.3)	1056 (55.5)
Race, No. (%)^a^						
Asian	15 (7.2)	18 (4.5)	29 (5.5)	18 (4.9)	20 (5.0)	100 (5.3)
Black	104 (50.0)	97 (24.1)	90 (17.1)	72 (19.7)	72 (17.9)	435 (22.9)
White	84 (40.4)	277 (68.7)	387 (73.7)	271 (74.2)	300 (74.6)	1319 (69.3)
Other or not provided^b^	5 (2.4)	11 (2.7)	19 (3.6)	4 (1.1)	10 (2.5)	49 (2.6)
Ethnicity, No. (%)^a^						
Hispanic or Latino	19 (9.1)	50 (12.4)	46 (8.8)	27 (7.4)	30 (7.5)	172 (9.0)
Not Hispanic or Latino	189 (90.9)	353 (87.6)	479 (91.2)	338 (92.6)	372 (92.5)	1731 (91.0)
BMI, mean (SD)	33.6 (4.7)	32.3 (4.2)	32.0 (4.4)	31.9 (4.5)	30.7 (4.2)	32.0 (4.4)
Physical activity (total MET h/wk), mean (SD)	112.5 (195.1)	115.1 (158.3)	104.9 (146.2)	105.3 (167.3)	113.7 (142.1)	109.8 (157.9)
Statin use, No. (%)	59 (28.4)	157 (39.0)	242 (46.1)	184 (50.4)	189 (47.0)	831 (43.7)
Personal vitamin D_3_ supplement use, No. (%)	33 (15.9)	144 (35.7)	268 (51.0)	176 (48.2)	214 (53.2)	835 (43.9)
Body weight, mean (SD), kg	99.2 (17.4)	93.9 (16.1)	93.5 (16.7)	93.8 (16.4)	87.0 (14.8)	92.9 (16.6)
Intratrial weight change, mean (SD), kg	0.5 (5.7)	−0.1 (5.2)	−0.0 (5.3)	−0.1 (5.8)	−0.8 (5.7)	−0.1 (5.5)
Serum 25(OH)D, mean (SD), ng/mL	16.0 (5.0)	24.2 (6.3)	29.8 (7.9)	30.1 (10.1)	35.0 (11.1)	28.3 (10.2)
Intratrial 25(OH)D, mean (SD), ng/mL	15.4 (3.3)	25.3 (2.9)	34.6 (2.8)	44.4 (2.8)	59.9 (8.2)	37.8 (15.0)
ApaI, No. (%)						
AA	69 (33.2)	134 (33.3)	147 (28.0)	92 (25.2)	118 (29.4)	560 (29.4)
AC	103 (49.5)	176 (43.7)	247 (47.0)	192 (52.6)	197 (49.0)	915 (48.1)
CC	32 (15.4)	88 (21.8)	124 (23.6)	78 (21.4)	85 (21.1)	407 (21.4)
Undetermined	4 (1.9)	5 (1.2)	7 (1.3)	3 (0.8)	2 (0.5)	21 (1.1)
BsmI, No. (%)						
CC	78 (37.5)	171 (42.4)	221 (42.1)	147 (40.3)	164 (40.8)	781 (41.0)
TC	102 (49.0)	177 (43.9)	230 (43.8)	177 (48.5)	181 (45.0)	867 (45.6)
TT	25 (12.0)	53 (13.2)	70 (13.3)	40 (11.0)	54 (13.4)	242 (12.7)
Undetermined	3 (1.4)	2 (0.5)	4 (0.8)	1 (0.3)	3 (0.7)	13 (0.7)
FokI, No. (%)						
AA	25 (12.0)	46 (11.4)	87 (16.6)	39 (10.7)	51 (12.7)	248 (13.0)
AG	83 (39.9)	188 (46.7)	216 (41.1)	162 (44.4)	184 (45.8)	833 (43.8)
GG	98 (47.1)	169 (41.9)	220 (41.9)	164 (44.9)	166 (41.3)	817 (42.9)
Undetermined	2 (1.0)	0	2 (0.4)	0	1 (0.2)	5 (0.3)

Abbreviations: BMI, body mass index (calculated as weight in kilograms divided by height in meters squared); MET, metabolic equivalent of task; VDR, vitamin D receptor.

SI conversion factor: To convert 25(OH)D to nanomoles per liter, multiply by 2.496.

^a^ Self-reported.

^b^ Included 9 American Indian or Alaska Native, 3 Native Hawaiian or Other Pacific Islander, and 37 specified only as “Other.”

The HRs and 95% CIs for incident diabetes across the intratrial 25(OH)D categories, overall and stratified by genotypes of ApaI, BsmI, and FokI, are shown in Table 2.^14^ For participants carrying the ApaI CC genotype, the HR was 0.29 (95% CI, 0.13-0.65) at 25(OH)D levels of 40 to 50 ng/mL and 0.17 (95% CI, 0.07-0.43) at levels of 50 ng/mL or more. For those carrying the AC genotype, the HR was 0.51 (95% CI, 0.30-0.86) at 25(OH)D levels of 40 to 50 ng/mL and 0.26 (95% CI, 0.14-0.48) at levels of 50 ng/mL or more. Participants with CC and AC genotypes were defined as responders. Participants with the AA genotype derived no risk reduction and were defined as nonresponders. A similar pattern was observed for BsmI; participants carrying 1 or 2 C alleles showed progressively lower risk of diabetes, whereas those with the TT genotype derived no risk reduction and were defined as nonresponders.

**Table 2.** Hazard Ratios for Incident Diabetes by VDR Genotype Within Each Intratrial Mean Serum 25(OH)D Category^a^

Allele	25(OH)D category, ng/mL									
	<20		20-29		30-39		40-49		≥50	
	HR (95% CI)	No.	HR (95% CI)	No.	HR (95% CI)	No.	HR (95% CI)	No.	HR (95% CI)	No.
ApaI (rs797523)										
CC	1.34 (0.51-3.54)	32	1 [Reference]	88	0.81 (0.45-1.46)	124	0.29 (0.13-0.65)	78	0.17 (0.07-0.43)	85
AC	0.86 (0.52-1.41)	103	1 [Reference]	176	0.76 (0.51-1.14)	247	0.51 (0.30-0.86)	192	0.26 (0.14-0.48)	197
AA	1.67 (0.84-3.31)	69	1 [Reference]	134	1.30 (0.75-2.27)	147	0.94 (0.46-1.93)	92	0.86 (0.42-1.77)	118
BsmI (rs1544410)										
CC	0.88 (0.48-1.60)	78	1 [Reference]	171	0.86 (0.57-1.30)	221	0.50 (0.29-0.84)	147	0.26 (0.14-0.49)	164
TC	1.45 (0.88-2.40)	102	1 [Reference]	177	0.91 (0.59-1.39)	230	0.59 (0.34-1.02)	177	0.44 (0.24-0.82)	181
TT	0.73 (0.15-3.61)	25	1 [Reference]	53	1.18 (0.50-2.79)	70	0.79 (0.26-2.40)	40	0.72 (0.22-2.32)	54
FokI (rs2228570)										
GG	1.89 (1.10-3.25)	98	1 [Reference]	169	1.36 (0.88-2.11)	220	0.87 (0.51-1.48)	164	0.57 (0.30-1.07)	166
AG	0.88 (0.49-1.56)	83	1 [Reference]	188	0.60 (0.40-0.92)	216	0.28 (0.16-0.48)	162	0.16 (0.09-0.30)	184
AA	1.13 (0.39-3.26)	25	1 [Reference]	46	1.03 (0.44-2.41)	87	0.55 (0.16-1.80)	39	0.40 (0.10-1.55)	51

Abbreviations: HR, hazard ratio; VDR, vitamin D receptor.

SI conversion factor: To convert 25(OH)D to nanomoles per liter, multiply by 2.496.

^a^ All analyses were adjusted for study site, assignment (vitamin D or placebo), race and ethnicity (Asian, Black, Hispanic or Latino, White, and other), and sex; and for baseline age, body mass index, usual physical activity, and statin use; and for weight change during the study. The 25(OH)D referent, 20 to 29.9 ng/mL, is the lower part of the adequate range of 20 to 50 ng/mL based on the National Academy of Medicine.^14^

For FokI, the pattern was less consistent (Table 2).^14^ The HR for the 162 participants with FokI AG with intratrial 25(OH)D levels of 40 to 50 ng/mL was 0.28 (95% CI, 0.16-0.48) and was 0.16 (95% CI, 0.09-0.30) for those with intratrial 25(OH)D levels of 50 ng/mL or more, while the associations for the GG and AA genotypes were not significant. Because of the lack of a graded discovery phase response of the FokI alleles, no further analyses of this polymorphism were performed.

### Test Analysis Phase

We examined the overlap between the alleles in the ApaI and BsmI polymorphisms among the 2086 participants with complete genotype data (eTable in Supplement 1). There was near-complete concordance among the nonresponders (ApaI AA and BsmI TT), which is representative of the degree of linkage disequilibrium observed in population studies. Specifically, 260 of 261 D2d study participants with the nonresponsive AA alleles of ApaI also had the nonresponsive TT alleles of BsmI.

The finding that participants carrying the ApaI AA genotype did not have reduced risk of type 2 diabetes at higher intratrial 25(OH)D levels, combined with the observation that 260 of 261 participants with the nonresponsive BsmI TT genotype also carried the ApaI AA genotype, suggested that knowing the ApaI genotype alone was sufficient to identify individuals with prediabetes who would be likely—or unlikely—to respond to high-dose vitamin D**_3_** supplementation.

In the test analyses, we analyzed all D2d participants with complete ApaI genotype data—the 2086 participants in the eTable in Supplement 1 plus an additional 12 participants with complete ApaI data but missing BsmI data. Participants (n = 2098; mean [SD] age, 60.2 [9.9] years; 1169 men [55.7%] and 929 women [44.3%]) were stratified solely by ApaI genotype into 2 groups: the proposed nonresponders to vitamin D**_3_**, consisting of those with the ApaI AA genotype (618 of 2098 [29.5%]), and the proposed responders to vitamin D**_3_**, consisting of those with the ApaI CC or AC genotypes (1480 of 2098 [70.5%]). The clinical characteristics of these 2 groups, by study treatment assignment (vitamin D**_3_** or placebo), are described in Table 3. These groups were generally well balanced at baseline. Both nonresponders and responders achieved similar intratrial serum 25(OH)D concentrations.

**Table 3.** Clinical Characteristics of Proposed Nonresponders and Responders by Assigned Treatment in the D2d Study^a^

Characteristic	Genetic variant group			
	Nonresponders (ApaI AA genotype)		Responders (ApaI AC and CC genotypes)	
	Vitamin D_3_ (n = 301)	Placebo (n = 317)	Vitamin D_3_ (n = 748)	Placebo (n = 732)
Age, mean (SD), y	59.3 (9.8)	59.8 (10.1)	59.9 (9.8)	61.1 (9.8)
Sex, No. (%)				
Female	128 (42.5)	143 (45.1)	334 (44.7)	324 (44.3)
Male	173 (57.5)	174 (54.9)	414 (55.3)	408 (55.7)
Race, No. (%)^b^				
Asian	7 (2.3)	8 (2.5)	50 (6.7)	47 (6.4)
Black	88 (29.2)	103 (32.5)	146 (19.5)	157 (21.4)
White	200 (66.4)	199 (62.8)	529 (70.7)	510 (69.7)
Other^c^	6 (2.0)	7 (2.2)	23 (3.1)	18 (2.5)
Ethnicity, No. (%)^b^				
Hispanic or Latino	30 (10.0)	22 (6.9)	77 (10.3)	65 (8.9)
Not Hispanic or Latino	271 (90.0)	295 (93.1)	671 (89.7)	667 (91.1)
BMI, mean (SD)	32.1 (4.6)	32.3 (4.3)	31.9 (4.4)	32.1 (4.5)
Physical activity, MET h/wk				
No. (No. missing)	293 (8)	313 (4)	735 (13)	720 (12)
Mean (SD)	124.1 (185.1)	127.0 (188.8)	105.1 (147.7)	105.7 (148.7)
Statin use, No. (%)	123 (40.9)	131 (41.3)	332 (44.4)	317 (43.3)
Personal vitamin D_3_ supplements, No. (%)	119 (39.5)	143 (45.1)	319 (42.6)	323 (44.1)
Baseline body weight, mean (SD), kg	94.2 (16.3)	94.2 (15.7)	92.6 (16.7)	92.9 (16.8)
Intratrial weight change, mean (SD), kg	−0.4 (5.4)	0.1 (5.3)	−0.2 (5.8)	−0.2 (5.1)
Baseline serum 25(OH)D, ng/mL				
No. (No. missing)	301 (0)	317 (0)	748 (0)	731 (1)
Mean (SD)	27.9 (11.1)	28.2 (10.7)	28.0 (9.9)	28.5 (9.8)
Intratrial achieved 25(OH)D, ng/mL				
No. (No. missing)	270 (31)	290 (27)	666 (82)	656 (76)
Mean (SD)	47.2 (14.3)	27.8 (9.9)	47.3 (13.1)	28.8 (9.5)

Abbreviations: BMI, body mass index (calculated as weight in kilograms divided by height in meters squared); MET, metabolic equivalent of task.

SI conversion factor: To convert 25(OH)D to nanomoles per liter, multiply by 2.496.

^a^ Based on the discovery analyses (see text), participants were classified as *potential nonresponders* (ApaI AAs) to vitamin D_3_ supplementation if they had alleles that were not associated with decreased risk of diabetes at higher achieved 25(OH)D levels. Conversely, participants were classified as *potential responders* (ApaI ACs and CCs) if they had alleles that showed reduced risk of diabetes at higher achieved 25(OH)D levels. This table is for descriptive purposes only; hence, no statistical tests were used to compare groups.

^b^ Self-reported.

^c^ Included 9 American Indian or Alaska Native, 3 Native Hawaiian or Other Pacific Islander, and 37 specified only as “other.”

Treatment with 4000 IU/d of vitamin D**_3_**, when compared with placebo, did not appear to reduce the risk of developing diabetes among the 618 participants with ApaI AA alleles (HR, 1.02 [95% CI, 0.72-1.44]). In contrast, among the 1480 participants with ApaI CC and AC alleles, 4000 IU/d of vitamin D**_3_** reduced the risk of developing diabetes by 19% (HR, 0.81 [95% CI, 0.66-0.99]). The timeline during which participants developed diabetes is shown in the Figure.

**Figure.**
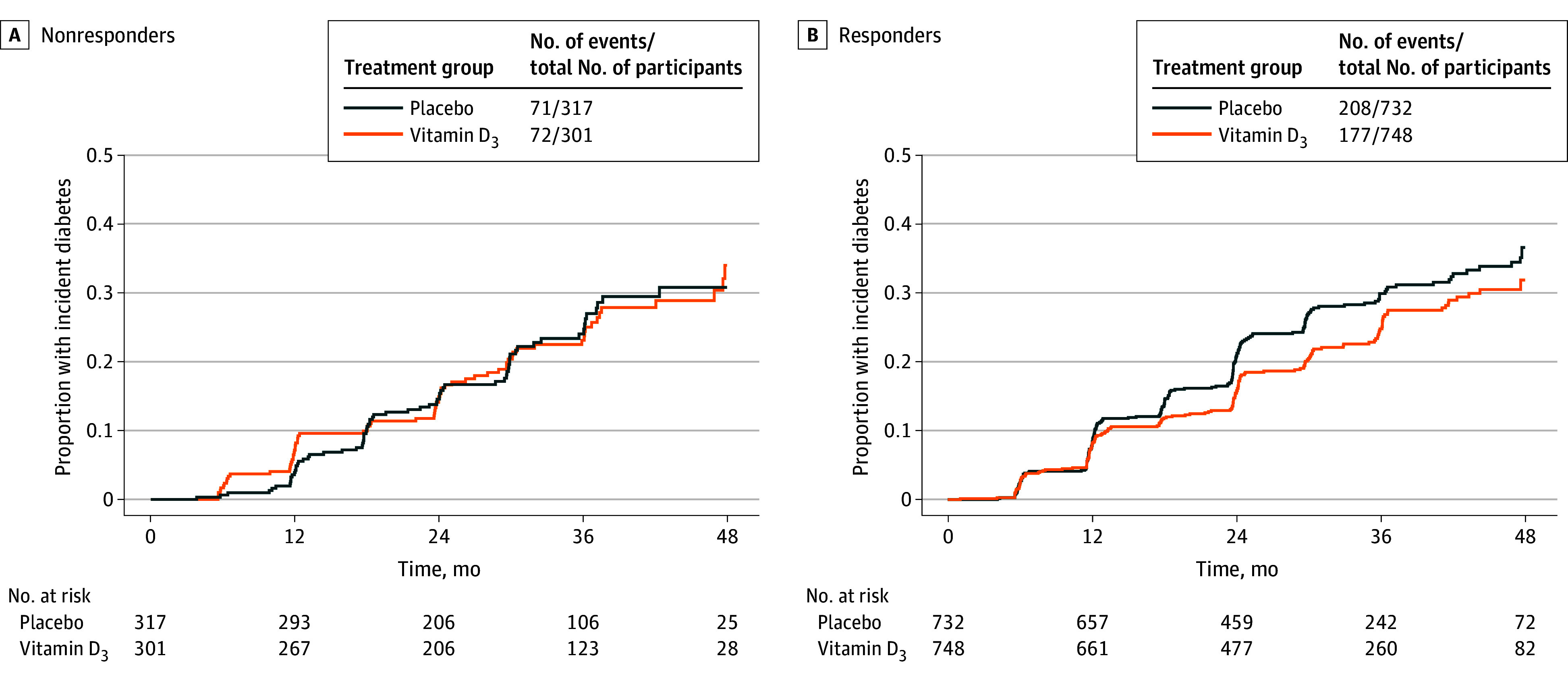
Proportions of Participants With Incident Diabetes, by Allele Status and Treatment Group A, Nonresponders with the ApaI AA allele. B, Responders with the ApaI AC and CC alleles.

## Discussion

This genetic association analysis of the D2d study suggests that genetic variation in the VDR, specifically the ApaI polymorphism, is associated with diabetes risk at higher intratrial 25(OH)D levels and is associated with response to 4000 IU/d of vitamin D_3_ supplementation among adults with prediabetes. Participants carrying the ApaI AA genotype did not experience a reduction in diabetes risk, either when achieving higher intratrial 25(OH)D concentrations or while being treated with 4000 IU/d of vitamin D_3_. In contrast, those carrying the ApaI CC and AC genotypes, representing 71% of the D2d study population, had progressively lower risk of type 2 diabetes at intratrial 25(OH)D levels of 40 ng/mL or higher. Participants with these genotypes randomized to vitamin D**_3_** had a 19% reduction in the risk of progression to diabetes compared with placebo, whereas those with ApaI AA alleles did not respond to treatment with vitamin D**_3_**. The BsmI polymorphism also appeared to play a role in the association between the achieved intratrial 25(OH)D level and diabetes risk, as expected given the high linkage disequilibrium of ApaI and BsmI (D’ = 1.0 and r^2^ = 1.0) among people of European ancestry.^15^ Because there was a near complete overlap between participants carrying the nonresponsive Bsml TT genotype and those carrying the nonresponsive ApaI AA genotype, knowing the ApaI genotype alone was sufficient to identify individuals who were likely—or unlikely—to respond to supplementation with 4000 IU/d of vitamin D**_3_**. These exploratory genetic association findings support our hypothesis that a common VDR variant modulates the link between high intratrial 25(OH)D levels and diabetes risk, and the association between relatively high-dose vitamin D**_3_** supplementation and diabetes risk among adults with prediabetes. The distributions of alleles of the 3 polymorphisms in the D2d study were similar to those reported in the UK Biobank of participants with prediabetes.^5^ Consistent with the UK Biobank study and other studies,^5,16,17^ the 25(OH)D levels achieved during the D2d trial did not differ significantly among participants with different VDR polymorphisms.

In the UK Biobank study, among adults with prediabetes and a median 25(OH)D level of 19.2 ng/mL (a value below our referent range of 20-29.9 ng/mL), there was a stepwise decrease in the risk of diabetes at 25(OH)D levels of lower than 10 (the study’s referent), 10 to 20, 20 to 30, and 30 ng/mL or higher.^5^ Risk reduction was present in all VDR genotypes of the 4 examined polymorphisms (ApaI, BsmI, TaqI, and FokI), but it was more prominent among those carrying the T allele of BsmI. There were too few participants in the D2d study with sufficiently low 25(OH)D levels to address this range of the 25(OH)D spectrum. Conversely, there were too few participants with sufficiently high 25(OH)D levels in the UK Biobank study to address the question posed in our study. To our knowledge, no other high-dose vitamin D trials among adults with prediabetes have examined how VDR polymorphisms may modify the effect of vitamin D supplementation on diabetes risk.

Our exploratory findings, if confirmed, hold promise for high-dose vitamin D**_3_** as a targeted, personalized approach to reducing the risk of type 2 diabetes among selected adults with prediabetes. The magnitude of the observed risk reduction among participants with AC and CC alleles of the ApaI polymorphism, if confirmed in an independent clinical trial, would have clinical implications for the management of prediabetes. In the original report of the D2d trial,^2^ the HR for conversion to type 2 diabetes with vitamin D supplementation was 0.88 (95% CI, 0.72-1.04). The HR decreased to 0.81 (95% CI, 0.66-0.99) in our exploratory analysis when genetically nonresponsive participants (those with AA alleles of the ApaI polymorphism, comprising 29.5% of all participants) were excluded. If confirmed, a 19% risk reduction in conversion to type 2 diabetes with vitamin D**_3_** supplementation would not be trivial. First, assessment of a single VDR polymorphism is inexpensive and now widely available. Second, many people could benefit because the global burden of prediabetes is large, involving an estimated 464 million individuals,^18^ and the prevalence is increasing. Preventing or slowing the transition from prediabetes to type 2 diabetes is a clinical priority because diabetes induces substantial worsening of multiple comorbidities, including microvascular disease, nephropathy, and retinopathy.^19,20^ Third, vitamin D supplementation is inexpensive, well tolerated, and safe, and adherence to a daily supplement does not involve the level of participant effort required to implement other lifestyle interventions known to reduce type 2 diabetes risk, such as sustained weight loss and regular exercise.^21^

### Strengths and Limitations

This study has several strengths. The D2d study had higher achieved 25(OH)D levels than in the UK Biobank, other cohort studies, and vitamin D intervention trials, owing to the relatively large vitamin D dose administered to half the participants and the allowance of 1000 IU per day of vitamin D for all participants. The D2d study used rigorous definitions of prediabetes and diabetes, based on intrastudy glycemic testing. In addition, the D2d study had frequent 25(OH)D measurements during the trial; therefore, a robust estimate of the intratrial 25(OH)D level was available for the discovery phase.

This study also has several limitations. Our study is too small to examine the association between ApaI alleles and treatment response within individual groups by race or ethnicity. An earlier study reported that each racial and ethnic group had a lower risk of diabetes at intratrial 25(OH)D levels of 40 ng/mL or higher, compared 20 with 29.9 ng/mL.^22^ Our study does not address the mechanisms by which ApaI genotypes are associated with the response to vitamin D. Given the post hoc nature of the gene association analyses, the observed effect modification of ApaI alleles on diabetes risk in response to vitamin D supplementation requires verification in a future vitamin D supplementation trial.

## Conclusion**s**

In this genetic association study of adults with prediabetes, diabetes risk reduction after supplementation with 4000 IU/d of vitamin D**_3_** was restricted to participants carrying the AC and CC alleles of the ApaI polymorphism. These findings support the potential use of ApaI genotyping to identify individuals most likely to experience a benefit from high-dose vitamin D treatment to reduce diabetes risk.

## Notes

Supplement 1. **eTable 1.** Distributions of ApaI and BsmI Alleles in Participants With Available Data in the ApaI and BsmI VDR Polymorphisms Showing High Overlap in the ApaI AA and BsmI TT Alleles **eTable 2.** Institutions That Approved the Study

Supplement 2.
Data Sharing Statement
