# Supplementary material for: Vitamin D Receptor Polymorphisms and the Effect of Vitamin D Supplementation on Diabetes Risk Among Adults With Prediabetes
Source: JAMA Netw Open. 2026 Apr 23;9(4):e267332. doi: 10.1001/jamanetworkopen.2026.7332 (PMC13107228; doi:10.1001/jamanetworkopen.2026.7332)
Supplement: Supplement 1. — eTable 1. Distributions of ApaI and BsmI Alleles in Participants With Available Data in the ApaI and BsmI VDR Polymorphisms Showing High Overlap in the ApaI AA and BsmI TT Alleles eTable 2. Institutions That Approved the Study [file jamanetwopen-e267332-s001.pdf]

# Supplementary Online Content

Dawson-Hughes B, Huggins GS, Nelson J, Vickery E, Powers SN, Pittas AG. Vitamin D receptor polymorphisms and the effect of vitamin D supplementation on diabetes risk among adults with prediabetes. *JAMA Netw Open.* 2026;9(4):e267332. doi:10.1001/jamanetworkopen.2026.7332

**eTable 1.** Distributions of ApaI and BsmI Alleles in Participants With Available Data in the ApaI and BsmI VDR Polymorphisms Showing High Overlap in the ApaI AA and BsmI TT Alleles

**eTable 2.** Institutions That Approved the Study

This supplementary material has been provided by the authors to give readers additional information about their work.

**eTable 1.** Distributions of ApaI and BsmI Alleles in Participants With Available Data in the ApaI and BsmI VDR Polymorphisms Showing High Overlap in the ApaI AA and BsmI TT Alleles

| | **ApaI CC** | **ApaI AC** | **ApaI AA** |
|---|---|---|---|
| **BsmI CC** | 455 | 346 | 75 |
| **BsmI CT** | 0 | 670 | 279 |
| **BsmI TT** | 0 | 1 | 260 |

© 2026 Dawson-Hughes B et al. *JAMA Network Open.*

**eTable 2.** Institutions That Approved the Study

| |
|---|
| Maine Medical Center |
| Tufts Medical Center |
| MedStar Health Research Institute |
| Duke University Medical Center |
| Medical University of South Carolina |
| Adventist Health System/Sunbelt Inc dba Florida Hospital |
| Atlanta VA Medical Center |
| Beth Israel Medical Center |
| Northwestern University |
| University of Tennessee Health Science Center |
| Tulane University |
| Pennington Biomedical Research Center |
| HealthPartners Research Foundation |
| University of Kansas Medical Center |
| Baylor College of Medicine |
| University of Nebraska Medical Center |
| University of Texas Southwestern Medical Center |
| University of Southern California |
| Stanford University Medical Center |
| Omaha VA Medical Center |
| MedStar Health Research Institute (Good Samaritan) |
| Kaiser Permanente |
| Cleveland Clinic |
| University of Colorado - Denver |
| Truman Medical Center (Satellite of University of Kansas Medical Center for recruitment and screening) |
| Denver VA Medical Center |
| Orlando VA Medical Center |
| Lenox Hill Hospital |

© 2026 Dawson-Hughes B et al. *JAMA Network Open.*
