# Supplementary material for: Vitamin D Receptor Polymorphisms and the Effect of Vitamin D Supplementation on Diabetes Risk Among Adults With Prediabetes
Source: JAMA Netw Open. 2026 Apr 23;9(4):e267332. doi: 10.1001/jamanetworkopen.2026.7332 (PMC13107228; doi:10.1001/jamanetworkopen.2026.7332)
Supplement: Supplement 2. — Data Sharing Statement [file jamanetwopen-e267332-s002.pdf]

Data Sharing Statement

Dawson-Hughes. Vitamin D Receptor Polymorphisms and the Effect of Vitamin D Supplementation on Diabetes Risk Among Adults With Prediabetes. *JAMA Netw Open*. Published April 23, 2026. doi:10.1001/jamanetworkopen.2026.7332

Data
Data available: Yes
Data types: Deidentified participant data
How to access data: bess.dawson-hughes@tufts.edu
When available: With publication

Supporting Documents
Document types: None

Additional Information
Who can access the data: The data will shared upon reasonable request at the discretion of the authors.
Types of analyses: For the purpose of studying the role vitamin D polymorphisms on the risk of type 2 diabetes at high serum 25-hydroxyvitamin D levels
Mechanisms of data availability: With investigator support after approval of a proposal
Any additional restrictions: None
